# ^18^F-FDG-PET/CT Whole-Body Imaging Lung Tumor Diagnostic Model: An Ensemble E-ResNet-NRC with Divided Sample Space

**DOI:** 10.1155/2021/8865237

**Published:** 2021-04-01

**Authors:** Zhou Tao, Huo Bing-qiang, Lu Huiling, Shi Hongbin, Yang Pengfei, Ding Hongsheng

**Affiliations:** ^1^School of Computer Science and Engineering, North Minzu University, Yinchuan 750021, China; ^2^Key Laboratory of Images & Graphics Intelligent Processing of State Ethnic Affairs Commission, North Minzu University, Yinchuan 750021, China; ^3^School of Science, Ningxia Medical University, Yinchuan 750004, China; ^4^Department of Urology, The General Hospital of Ningxia Medical University, Yinchuan 750004, China; ^5^Department of Nuclear Medicine, The General Hospital of Ningxia Medical University, Yinchuan 750004, China

## Abstract

Under the background of ^18^F-FDG-PET/CT multimodal whole-body imaging for lung tumor diagnosis, for the problems of network degradation and high dimension features during convolutional neural network (CNN) training, beginning with the perspective of dividing sample space, an E-ResNet-NRC (ensemble ResNet nonnegative representation classifier) model is proposed in this paper. The model includes the following steps: (1) Parameters of a pretrained ResNet model are initialized using transfer learning. (2) Samples are divided into three different sample spaces (CT, PET, and PET/CT) based on the differences in multimodal medical images PET/CT, and ROI of the lesion was extracted. (3) The ResNet neural network was used to extract ROI features and obtain feature vectors. (4) Individual classifier ResNet-NRC was constructed with nonnegative representation NRC at a fully connected layer. (5) Ensemble classifier E-ResNet-NRC was constructed using the “relative majority voting method.” Finally, two network models, AlexNet and ResNet-50, and three classification algorithms, nearest neighbor classification algorithm (NNC), softmax, and nonnegative representation classification algorithm (NRC), were combined to compare with the E-ResNet-NRC model in this paper. The experimental results show that the overall classification performance of the Ensemble E-ResNet-NRC model is better than the individual ResNet-NRC, and specificity and sensitivity are more higher; the E-ResNet-NRC has better robustness and generalization ability.

## 1. Introduction

Lung tumors [1, 2] are one of the malignant tumors with high morbidity and mortality [3]. The data reveals that the incidence of lung tumors is increasing year by year, which is a serious threat to human health. The early clinical features of lung tumors are pulmonary nodules [4]. There are no specific clinical symptoms; hence, it is difficult to be detected and diagnosed in time. Once the disease is diagnosed, the cancer is at an advanced stage. Therefore, early diagnosis and early detection are essential for the treatment of lung cancer. Medical imaging techniques [5, 6] are widely used in the diagnosis of lung tumors, such as ultrasound, X-ray imaging, Computerized Tomography imaging(CT), Magnetic Resonance Imaging(MRI), and positron emission tomography imaging(PET). In particular, the advantages of PET and CT are combined by ^18^F-FDG-PET/CT [7]. It can realize the same machine fusion of anatomical image CT and functional metabolism image PET and accurately locate physical characteristics of the lesion, such as the location, size, shape, and density of the lesion. Finally, the effect of “1 + 1 > 2” is achieved. Mass medical images not only provide more detailed and accurate diagnostic information but also increase the workload of clinicians. Computer-aided diagnosis system (CAD) for lung tumors is an effective solution [8, 9]. On the one hand, CAD can provide doctors with accurate quantitative analysis services, so as to make up for the defects of human inertia and insensitivity to gray scales [10, 11]; on the other hand, it can effectively reduce the error rate of doctors' interpretation of medical images, thereby helping doctors to better diagnose diseases and improve the diagnosis rate.

Ensemble learning is a machine learning paradigm. Its essence is to use multiple classifiers to solve the same problem and finally use “majority voting” to determine the final result [12]. In recent years, deep learning has become a machine learning hot topic. It has been successfully applied in the field of medical image processing, especially in the auxiliary classification, recognition, detection, and segmentation of malignant tumors, achieving impressive results that surpass human performance. Ensemble deep learning, which couples deep learning and ensemble learning, can make full use of the advantages of the two methods and can provide a new research direction for computer-aided diagnosis. For example, Wang et al. employed transfer learning with relative majority voting to construct a convolutional neural network (CNN) model for the computer-aided diagnosis of lung tumors [13]. In another work, Xiao et al. [14] ensemble a variety of different machine learning models for the accurate diagnosis of lung cancer; five classifiers, namely, k-nearest neighbor (KNN), support vector machine (SVM), decision trees (DTs), random forest (RF), and gradient boosted decision tree (GBDTs), were ensembled to construct a multimodal ensemble model to predict the incidence of both normal and abnormal cancer. Harangi [15] uses an integrated method to integrate four types of deep neural networks, including AlexNet, GoogleNet, VGG and ResNet. Yu and Wang [16] integrated the three deep learning network models of AlexNet, GoogleNet, and VGG for computer-aided diagnosis of lung cancer; there are good generalization ability of ensemble network model. Alzubi and Bharathikannan [17] use weight optimization and maximum likelihood boosting (MLB) to achieve a better false-positive rate and accuracy. Sirazitdinov et al. [18], propose an ensemble of two convolutional neural networks, namely RetinaNet and Mask R-CNN for pneumonia detection and localization. The algorithm is validated on a recently released dataset of 26,684 images from the Kaggle Pneumonia Detection Challenge and scored among the top 3% of submitted solutions.

R-Ensembler, a parameter free greedy ensemble attribute selection method is proposed by Bania and Halder [19] adopting the concept of rough set theory by using the attribute-class, attribute-significance and attribute-attribute relevance measures to select a subset of attributes which are most relevant, significant and non-redundant from a pool of different attribute subsets in order to predict the presence or absence of different diseases in medical dataset. The main role of the proposed ensembler is to combine multiple subsets of attributes produced by different rough set filters and to produce an optimal subset of attributes for subsequent classification task. Cao et al. [20] propose an ensemble ELM (Extreme Learning Machine) combining with the SRC (En-SRC) algorithm. Rather than using the output vector from single ELM to decide the threshold for data partition, En-SRC incorporates multiple ensemble outputs to improve the reliability and classification accuracy. Jiang et al. [21] propose a contextual attention mechanism and a spatial attention mechanism for learning fine-grained representation of pulmonary nodules. an ensemble of 3D Dual Path Networks (DPNs) is used to boost the pulmonary nodule classification performance, Experimental results demonstrate the effectiveness of the proposed method.

Improving the generalizability of individual classifiers and increasing the heterogeneity of individual classifiers in ensemble learning architectures are two crucial factors that can improve the performance of ensemble learning models. Therefore, from the perspective of splitting the sample space in the framework of ensemble learning, and based on the ResNet with nonnegative representation classification (NRC), a ^18^F-FDG-PET/CT whole-body imaging lung tumor diagnosis E-ResNet-NRC model is proposed. Firstly, three modalities of PET, CT, and PET/CT medical images of lung tumors are collected; according to the medical image modality, the medical image is divided into three sample spaces: PET, CT, and PET/CT. Secondly, constructing an individual classifier based on residual neural network in a different sample space, each individual classifier is trained by migration learning, which can ensure the rapid learning ability of the individual classifier and the difference of the individual classifier. Thirdly, using nonnegative representation classification NRC in the fully connected layer improves the sparse representation ability and classification performance of sample data. Finally, a relatively majority vote is used for ensemble learning, and the results of computer-aided diagnosis of lung tumor images are obtained.

## 2. Background

### 2.1. ^18^F-FDG-PET/CT Whole-Body Imaging

**Figure 1:**
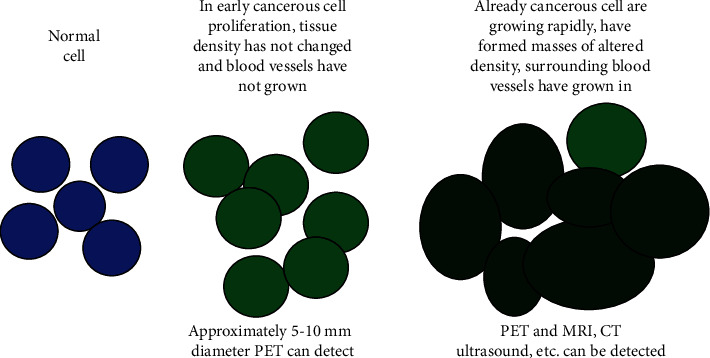
Molecular imaging of tumor cells.

Molecular imaging is a science that uses imaging techniques to reveal the distinct levels of tissue organization at the cellular and subcellular levels, reflecting variations in vivo at the molecular level and allowing to conduct qualitative and quantitative research on biological behaviors based on images. ^18^F-FDG-PET/CT is an important assessment modality in molecular imaging. It can detect the initial state of the disease in the body before the disease shows clinical symptoms or changes in anatomical structure. Therefore, early intervention of the disease can be realized, and the purpose of reversing, preventing or delaying the occurrence of the disease can be achieved, and the efficiency of the disease cure can be greatly improved (Figure 1).

### 2.2. ResNet

**Figure 2:**
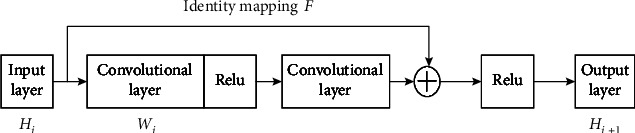
Residual block.

The ResNet(ResiDual Neural network) is composed of convolutional layers for feature extraction and pooling layers for feature processing. After multiple convolution and pooling operations, the input image is classified and output through a fully connected layer [22]. The ResNet uses shortcut connections and fitting residual representations. The identity mapping reconstructs the learning process, redirects the network information flow, and increases the depth of the network. This improves the representation capability of the model, accelerates the network convergence speed, and effectively solves common issues such as network degradation and gradient vanishing. The residual neural network is composed of multiple residual block structures overlapping, while adjacent convolutional layers are connected by shortcuts to form residual blocks. The structure of the residual block is shown in Figure 2.

*H*_*i*_ represents the input, *H*_*i*+1_ represents the output, *W*_*i*_ represents the weights, and *F* represents residual mapping. The residual block mapping is thus represented as follows:

(1) $$\begin{array}{c} H_{i+1}=\text{Relu}\left(H_{i}+F\left(H_{i}W_{i}\right)\right). \end{array}$$

When the input dimension *H*_*i*_ and the output dimension *H*_*i*+1_ are different, the linear projection *φ* is used to match the dimensions. Therefore, Equation (1) can be expressed as

(2) $$\begin{array}{c} H_{i+1}=\text{Relu}\left(\varphi {\left(H\right)}_{i}+F\left(H_{i}W_{i}\right)\right). \end{array}$$

The residual mapping is more easily learned empirically through experimentation when compared with the original mapping. Therefore, the ResNet learns the residual mapping through the middle stacked layers. The residual mapping *F* is more sensitive to variations in the output, and the parameter adjustment range is comparably broader, thus speeding up learning and improving the network optimization performance. Therefore, the ResNet-50 network was chosen in this study.

### 2.3. NRC Algorithm

In recent years, sparse representation [23, 24] of high-dimensional feature data has become a research hot topic in the field of machine learning. Sparse representation classification (SRC) [25, 26] for high-dimensional data recognition proves advantageous in improving sparse representation and classification performance. The main concept of SRC is the association of the test sample with a linear combination of the training samples; then, the test samples are divided into their corresponding classes with the minimum distance or approximation error [27]. However, the encoding coefficient of SRC is negative, which, in practice, causes the weights corresponding to the positive and negative coefficients to offset. This affects the classification accuracy to some extent. The classification criterion of nonnegative representation classification (NRC) [28] is the classification according to the similarity of training and test samples. This approach is similar to sparse representation classification (SRC) with the difference being that the coding coefficient of NRC is limited to nonnegative [29]. The nonnegative representation can improve the representation of isomorphic samples while inhibiting the representation of heterogeneous samples, resulting in sparse encoding coefficients from the same correct class; therefore, the nonnegative representation is at the same time sparse and distinguished. Therefore, nonnegative representation tends to find homogeneous samples, which translates to higher recognition accuracy [30].

The main idea of NRC revolves around the query samples *y* ∈ **R**^**D**^, and the training sample matrix $X=\left[X_{1},\cdots .,X_{k}\right]\in R^{D\times N}$. Firstly, each column of *Y* and *X* is normalized to a unit *L*_2_ standard; the encoding vector $\hat{c}$ is then calculated by querying the samples *Y* and *X*. The larger the difference between reconstruction residuals, as calculated from the matric coefficients, the higher the similarity of the test sample to the training sample. The output label category is assigned based on the degree of residual similarity. The algorithm design is shown in Table 1.

### 2.4. Ensemble Learning

The core concept of ensemble learning is to train multiple homogeneous and different individual learning algorithms to solve the same problem [31]. Then, the final predicted result is obtained by combining the weighed outputs of all individual learners through a variety of strategies. In order to design a robust ensemble classification model, it is necessary to improve the generalization ability of individual classifiers as well as to increase the differences between the individual classifiers in the ensemble.

Ensemble learning [31] can significantly improve the generalization ability of the learning system. The most common techniques include bagging, boosting, and stacking. The conventional methods used to generate base classifiers can be roughly divided into two broad categories: the first one comprising the application of different types of learning algorithms to the same data set, with the resulting base classifier referred to as heterogeneous, and the second one consisting on the application of the same learning algorithm to different training sets, producing a homogeneous classifier [32].

The combination of strategies of ensemble learning for classifiers includes the average, voting, and learning methods. Different combinations of methods are chosen depending on the application. For example, for regression estimation, the prediction results of individual learners are usually simply averaged or weighed averaged. Meanwhile, for classification, the results of each individual classifier are usually voted to obtain the final classification result. The voting method is divided into the absolute majority voting and the relative majority voting method. The absolute majority voting method is characterized by more than half of the individual learners delivering the same answer; the output is the final classification result of the ensemble. The relative majority voting method is characterized by the majority of individual learners outputting a certain classification result, this result is the final classification result of the ensemble.

## 3. Ensemble E-ResNet-NRC Model with Partitioned Sample Space

### 3.1. Algorithm Rationale

In this study, an ensemble E-ResNet-NRC model with partitioned sample space is proposed. The overall design of the model is as follows:

#### 3.1.1. Data Collection

**Figure 3:**
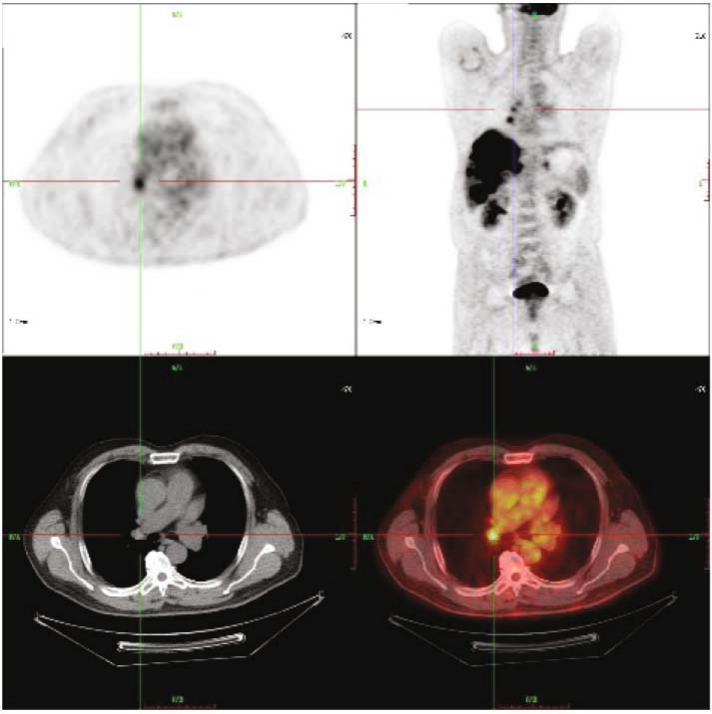
CT, PET, and PET/CT original images.

9000 CT, PET, and PET/CT of patient's lung images were collected from a 3A hospital in Ningxia between 2014 and 2016, including 3000 cases of each modal image.  Figure 3 shows a PET image of lung tumor (upper left), CT image of a pulmonary tumor (lower left), whole-body image (upper right), and PET/CT image of lung tumor (lower right).

*Sample Set Division*. Lung medical image sample set is as follows: Sample_Lung, Sample Size  | Sample_Lung | = 9000, according to the types of medical image (CT, PET, or PET/CT). Sample_Lung = {Sample_CT, Sample_PET, Sample_PET/CT}. The sample lung was divided into three sample subsets: Sample_CT, Sample_PET, and Sample_PET/CT. with sample sizes ∣Sample_CT | = 3000, ∣Sample_PET | = 3000, and ∣Sample_PET/CT | = 3000. The negative and positive samples of each sample subset are the same, i.e., Sample_CT = {Sample_CT_Negative Sample_CT_Positive}, ∣Sample_CT_Negative | = ∣Sample_CT_Positive | = 1500, Sample_PET = {Sample_PET_Negative Sample_PET_Positive}, ∣Sample_PET_Negative | = ∣Sample_PET_Positive | = 1500, Sample_PET/CT = {Sample_PET/CT_Negative Sample_PET/CT_Positive}, ∣Sample_PET/CT_Negative | = ∣Sample_PET/CT_Positive | = 1500.

#### 3.1.2. Transforming Pseudocolor into Gray Images

Sample_Lung = rgb2gra(Sample_Lung).

#### 3.1.3. ROI

Local features (i.e., the region of interest) are extracted from the global gray images based on clinical markers corresponding to the lesion area. Then, the ROI is normalized to experimental data as 50px × 50px, sample lung = ROI lung (sample lung). The ROI extraction process for each of the three sample subsets is as follows: Sample_CT_ROI = ROI_Lung(Sample_CT), Sample_PET_ROI = ROI_Lung(Sample_PET), Sample_PET/CT_ROI = ROI_Lung(Sample_PET/CT).

#### 3.1.4. Constructing Different Sample Spaces

The lung medical image sample set (Sample_Lung) is composed of three different medical image modalities CT, PET, and PET/CT. The local features of the lesion area are used to define the ROI and obtain the same set as the original Sample_CT_ROI: Sample_CT_ROI = {Sample_CT_ROI, Sample_PET_ROI, Sample_PET/CT_ROI}. In these three sample subsets, each of 3000 cases and each sample subset (negative and positive samples) are the same size, i.e., 1500 cases: Sample_CT_ROI = {Sample_CT_ROI − Negative Sample_CT_ROI_Positive}, ∣Sample_CT_ROI_Negative | = ∣Sample_CT_ROI_Positive | = 1500, Sample_PET_ROI = {Sample_PET_ROI_Negative Sample_PET_ROI_Positive}, ∣Sample_PET_ROI_Negative | = ∣Sample_PET_ROI_Positive | = 1500, Sample_PET/CT_ROI = {Sample_PET/CT_ROI_Negative Sample_PET/CT_ROI_Positive}, ∣Sample_PET/CT_ROI_Negative | = ∣Sample_PET/CT_ROI_Positive | = 1500.

**Figure 4:**
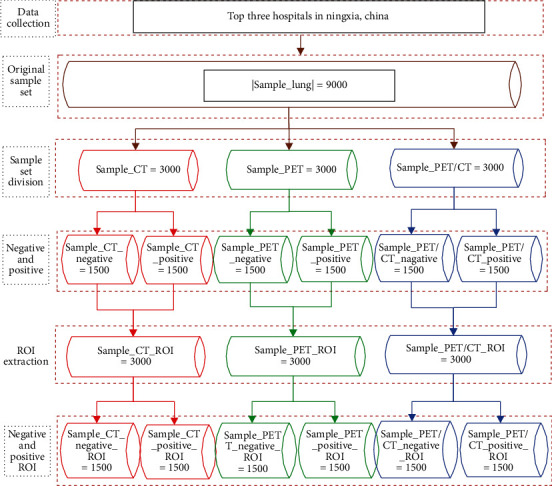
Sample_Lung set division.

Figure 4 shows that Sample_Lung set is divided into three sample spaces.

#### 3.1.5. Construction of a Fivefold Cross-Experimental Dataset Based on Sample Space Division in the Three Sample Subsets, Namely Sample_CT_ROI, Sample_PET_ROI, and Sample_PET_CT_ROI

A dividing algorithm was used to separate the negative and positive sample sets of each sample subset into 5 uniform datasets, each one of 300 samples, to obtain a 5-fold cross-sample set.

#### 3.1.6. Construction of the ResNet-NRC

Individual classifiers were designed based on sample subsets of the three image modalities.

1. The ResNet-50 was pretrained via transfer learning. The parameters in the pretraining network were taken as the initialization parameters: ResNet − NRC = Transfer learning (ResNet-50, NRC); Table 2 shows ResNet-50 parameters

(2) In three sample subsets Sample_CT_ROI, Sample_PET_ROI, and Sample_PET/CT_ROI, the ResNet-NRC network is retrained to get individual classifiers: ResNet − NRC − CT = Training(ResNet − NRC, Sample_CT_ROI), ResNet − NRC − PET = Training(ResNet − NRC, Sample_PET_ROI), ResNet − NRC − PET/CT = Training(ResNet − NRC, Sample_PET/CT_ROI).

#### 3.1.7. The ResNet-NRC Classifier

The ResNet-NRC classifier was ensembled via relative majority voting to obtain three individual classifiers: ResNet − NRC = Ensemble{(ResNet − NRC − CT, ResNet − NRC − PET, ResNet − NRC − PET}.

**Figure 5:**
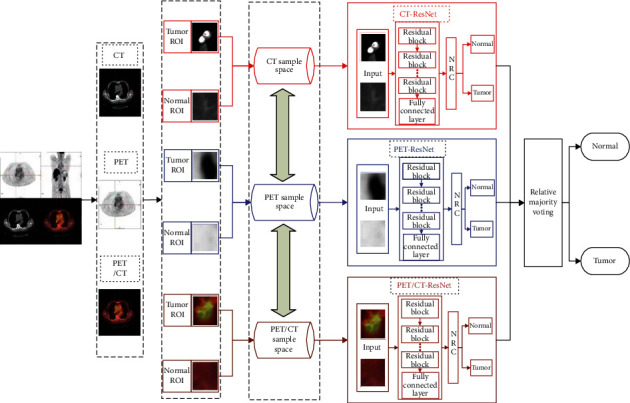
Algorithm flow chart of ensemble E-ResNet-NRC.

Figure 5 shows an algorithm flow chart.

### 3.2. Key Technology: ResNet-NRC Model

Transfer learning refers to the initialization of a small training set of parameters by using a pretrained network with a proven learning capacity. This method can this be used to transfer existing learning abilities from one network to another. In this paper, three individual classifiers, namely, ResNet-NRC-CT in CT mode, ResNet-NRC-PET in PET mode, and ResNet-NRC-PET/CT in PET/CT mode, were constructed via transfer learning based on the ResNet-50. This model was used to identify lung tumors from CT, PET, and PET/CT medical images, respectively.

Input: The three sample subsets Sample_CT_ROI, Sample_PET_ROI, and Sample_PET/CT_ROI.

Output: Three ResNet-NRC Individual classifiers, ResNet-NRC-CT, ResNet-NRC-PET, and ResNet-NRC-PET/CT.

The process to obtain these is as follows.

(1) Transfer learning was used to train the ResNet-50: ResNet = TransferLearning(ResNet − 50)
(2) For the three modalities, the initialization parameters are taken from the pretrained ResNet-50 network. The training and extraction of the fully connected layer features carried in the ResNet: ResNet − CT = Training(ResNet, Sample_*i*_ROI), *i* = 1, 2, 3, where 1, 2, and 3 refer to CT, PET, and PET/CT, respectively
(3) Taking the Sample-CT-ROI as an example, for the training samples *X* = [*X*_1_, ⋯*X*_*k*_], *X*_*i*_∈ Sample-CT-ROI, and testing sample *y* = [*y*_1_, ⋯*y*_*n*_], *y*_*i*_∈ Sample-CT-ROI. Through the ResNet-50 feature extraction, the training sample matrix of the feature space is obtained as *X*′ = [*X*_1_′, ⋯*X*_*k*_′], with a test sample matrix *y*′ = [*y*_1_′, ⋯*y*_*n*_′].
(4) Each column of the matrix *X*′ and query sample *y*′ are normalized to unit *L*_2_ standard:
  (3) $$\begin{array}{c} {\left\|X^{\prime }\right\|}_{2}=\sqrt{\overset{n}{\underset{i=1}{\sum }}{x^{\prime }}_{i}^{2}},\\ {\left\|y^{\prime }\right\|}_{2}=\sqrt{\overset{n}{\underset{i=1}{\sum }}{y^{\prime }}_{i}^{2}} \end{array}$$
(5) The training sample *X*′ in the feature space *y*′ is nonnegative. Therefore, the nonnegative coefficient $\hat{c}$ can be obtained as
  (4) $$\begin{array}{c} \hat{c}=\arg\min_{\text{c}}\left\|y^{\prime }-X^{\prime }c\right\|\text{s}.\text{t}.c\geq 0 \end{array}$$
(6) Training samples are used to classify the nonnegative representations of the test samples based on their similarity as
  (5) $$\begin{array}{c} r_{k}={\left\|y^{\prime }-X_{k}^{\prime }{\hat{c}}_{k}\right\|}_{2} \end{array}$$
(7) Finally, the label category of the residual output result is defined as
  (6) $$\begin{array}{c} \text{Label}\left(y^{\prime }\right)=\arg\min\left\{r_{k}\right\}. \end{array}$$

## 4. Experiments

### 4.1. Experimental Environment

Software environment is as follows: Windows10 operating system, MatlabR2019a; hardware environment is as follows: Intel(R)Core(TM)i5-7200U CPU @2.50GHz 2.70GHz, 4.0GB memory, 500GB hard drive.

### 4.2. Evaluation Metrics

In this paper, the evaluation metrics include accuracy, sensitivity, specificity, *F*-score value, and Matthews correlation coefficient (MCC), which are described as follows:

Accuracy, sensitivity, and specificity were calculated by true-positive (TP), false-positive (FP), true-negative (TN), and false-negative (FN). TP indicates a benign tumor was predicted correctly, FP indicates a malignant tumor was predicted incorrectly, TN indicates a malignant image was predicted correctly, and FN indicates that benign tumors were predicted incorrectly. They are calculated by the following formulae:

(7) $$\begin{array}{c} \text{Accuracy}=\frac{\text{TP}+\text{TN}}{\text{TP}+\text{TN}+\text{FP}+\text{FN}},\\ \text{Sensitivity}=\frac{\text{TP}}{\text{TP}+\text{FN}},\\ \text{Specificity}=\frac{\text{TN}}{\text{TN}+\text{FP}}. \end{array}$$

The *F*-value is a summed average of the percentages of completeness and accuracy. It is used as a trade-off between accuracy and recall. The calculation formula is as follows:

(8) $$\begin{array}{c} F=\frac{2\times \text{TP}}{2\times \text{TP}+\text{FP}+\text{FN}}. \end{array}$$

MCC is a more comprehensive evaluation metric that reflects the reliability of the algorithm. When the number of categories is different, the value of the measure is considered balanced ranging from -1 to +1. The MCC takes the value of 1 when the prediction error is 0 for both FP and FN, which means that the classification is completely correct; when the prediction error is 0 for both TP and TN, the MCC takes the value of -1, which means that the classification is completely wrong. It is calculated as follows:

(9) $$\begin{array}{c} \text{MCC}=\frac{\text{TP}\times \text{TN}‐\text{FP}\times \text{FN}}{\sqrt{\left(\text{TP}+\text{FP}\right)\left(\text{TP}+\text{FN}\right)\left(\text{TN}+\text{FP}\right)\left(\text{TN}+\text{FN}\right)}}. \end{array}$$

### 4.3. Experimental Results and Analysis

The experiments were performed using a 5-fold cross-validation for training. The final results were averaged over five experiments. 2400 training samples and 600 test samples were used. The experiments were carried out in CT, PET, and PET/CT trimodal datasets. AlexNet and ResNet-50 were used for comparison. Classification was achieved through the nearest neighbor classification (NNC), softmax, and nonnegative representation classification (NRC) algorithms. The algorithms were pairings were as follows: AlexNet+NNC, AlexNet+Softmax, AlexNet+NRC, ResNet-50+NNC, ResNet-50+Softmax, and ResNet-50+NRC.

#### 4.3.1. Experiment 1: Comparison of the Accuracy and Times of the Different Models

This experiment explored the effects of different network models, classification algorithms, and sample spaces on the ResNet recognition rate and training time. The following six combinations of algorithms were examined: AlexNet+NNC, AlexNet+Softmax, AlexNet+NRC, ResNet-50+NNC, ResNet-50+Softmax, and ResNet-50+NRC. The recognition accuracy, running time for training, and standard deviation (SD) in the sample space of CT, PET, and PET/CT are shown in Table 3.

*(1) Not Using Ensemble Learning*. The experiment was also carried out without using ensemble learning. In the first scenario, different network models with the same classification algorithms were used. As in Experiment 1, three groups of comparative experiments were performed, namely, AlexNet+NNC and ResNet-50+NNC, AlexNet+Softmax, and ResNet-50+Softmax, as well as AlexNet+NRC and ResNet-50+NRC.

Taking the third group as an example, in the CT sample space, the accuracy of the proposed ResNet-50+NRC model was 0.27% higher than that of the AlexNet+NRC model with a training time of 1019.04 seconds. It is noted that the ResNet is deeper when compared with the AlexNet; for this reason, the extracted image features are richer, and the classification accuracy is higher; however, the training time is greatly increased, in this case, by 648.14%. The results of the other two groups were similar (data not shown).

In the second scenario, the same network with different classification algorithms was used. In Experiment 1, there were three groups of comparative experiments, namely, AlexNet+NNC and AlexNet+Softmax, AlexNet+NRC and ResNet-50+NNC, and ResNet-50+Softmax and ResNet-50+NRC. Taking the second group as an example, in the CT sample space, the classification accuracy of the individual classifier ResNet-50+NRC was 2.07% and 0.94% higher than that of the ResNet-50+NNC and ResNet-50+Softmax, respectively. In terms of the training times, Net-50+NRC was 28.04 and 22.67 seconds faster than the ResNet-50+NNC and ResNet-50+Softmax models, respectively. Compared with the first scenario, the overall training time was greatly improved; however, after the network model was determined, the increase in training time was not significant. It is noted that when using the same network architecture, the NRC model exhibits a better classification accuracy when compared with the NNC and Softmax models. This algorithm also proved suitable for handling high-dimensional data and reduced training times significantly.

*(2) Using Ensemble Learning*. In this experiment, the same network architecture and classification algorithms under different sample spaces were used. Six groups of comparative experiments were considered: AlexNet+NNC and E-AlexNet+NNC, AlexNet+Softmax and E-AlexNet+Softmax, AlexNet+NRC and E-AlexNet+NRC, ResNet-50+NNC and E-ResNet-50+NNC, ResNet-50+Softmax and E-ResNet-50+Softmax, and ResNet-50+NRC and E-ResNet-50+NRC.

Taking the third group in the three different sample spaces as an example, the classification accuracy of E-AlexNet+NRC model was 0.63% and 1.46% higher than that of the AlexNet+NRC in the CT and PET/CT sample spaces, respectively. When taking the sixth group in the three sample spaces as an example, the classification accuracy of the proposed E-ResNet-50+NRC model was 0.50% and 1.24% higher than that of the ResNet-50+NRC model in the sample space of CT and PET/CT, respectively. Meanwhile, the training time was improved by 1974.43 and 1992.16 seconds, respectively. It is noted that when using the same network model and classification algorithm on different sample spaces, ensemble learning can improve the classification accuracy at the expense of substantially increased training times. From the comparative experiments in Experiment 1, namely, E-AlexNet+NNC, E-AlexNet+Softmax, E-AlexNet+NRC, E-ResNet-50+NNC, E-ResNet-50+Softmax, and E-ResNet-50+NRC, the classification accuracy of the proposed E-ResNet-50+NRC model was 99.57%—the highest among the six tested models.

#### 4.3.2. Experiment 2: Comparison of Evaluation Indexes of Different Models

In this experiment, six algorithms were examined: AlexNet+NNC, AlexNet+Softmax, AlexNet+NRC, ResNet-50+NN, ResNet-50+Softmax, and ResNet-50+NRC. Training and recognition were carried out in three sample spaces: CT, PET, and PET/CT. The algorithms were evaluated in terms of their accuracy, sensitivity, specificity, *F*-value, and MCC (Tables 4–8).

**Figure 6:**
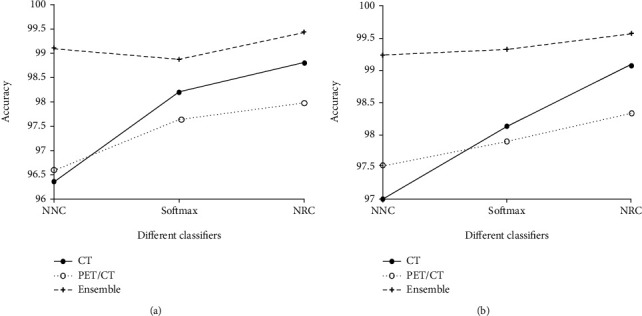
Accuracy of the AlexNet and ResNet-50 models. (a) Accuracy of the AlexNet model. (b) Accuracy of the ResNet-50 model.

**Figure 7:**
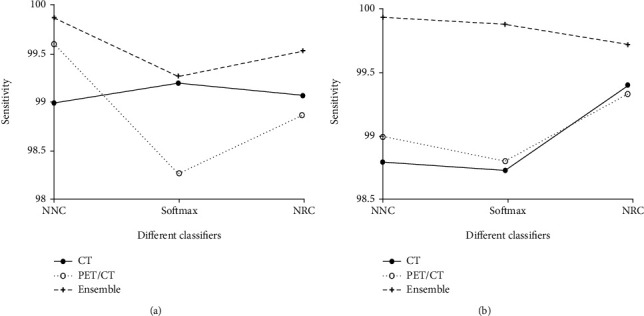
Sensitivity of the AlexNet and ResNet-50 models. (a) Sensitivity of the AlexNet model. (b) Sensitivity of the ResNet-50 model.

**Figure 8:**
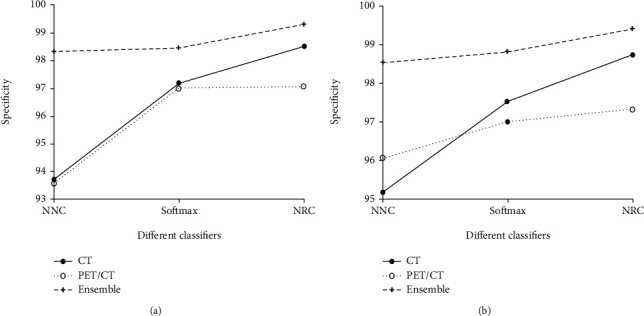
Specificity of the AlexNet and ResNet-50 models. (a) Specificity of the AlexNet model. (b) Specificity of the ResNet-50 model.

**Figure 9:**
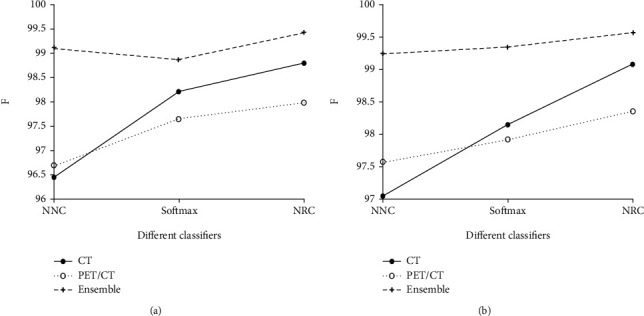
*F*-score of the AlexNet and ResNet-50 models. (a) *F*-score of the AlexNet model. (b) *F*-score of the ResNet-50 model.

**Figure 10:**
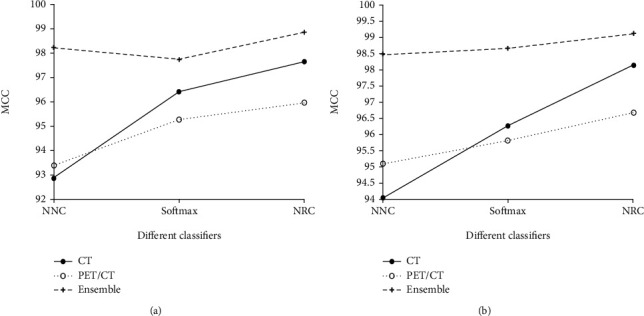
MCC of the AlexNet and ResNet-50 models. (a) MCC of the AlexNet and ResNet-50 models. (b) MCC of the AlexNet and ResNet-50 models.

From Tables 4–8, it is noted that when using different network architectures with the same classification algorithm, the ResNet50-NRC showed improvements of 0.27%, 0.33%, 0.2%, 0.13%, and 0.48 seconds in accuracy, sensitivity, specificity, *F*-value, and MCC, respectively, when compared with the AlexNet-NRC and the Text E-ResNet50-NRC. When compared with the AlexNet-NRC, the sensitivity, specificity, *F*-value, and MCC were increased by 0.14%, 0.2%, 0.07%, 0.14%, and 0.36%, respectively. Plotting the average value of the indicators presented in Figures 6–10 provides with a clear visual representation of the differences between the different algorithms.

From the information derived from the above experiments and analyses, it is noted that, when using the same network architecture, the NRC algorithm exhibited a better performance when compared with the NNC and Softmax algorithms. The NCR algorithm with a ResNet proved more robust for handling high-dimensional data in the CT, PET, and PET/CT sample spaces. In terms of classification accuracy, the experimental results showed that the ResNet-50 architecture was better suited when compared with the AlexNet. The ResNet reconstructs the learning process and redirects the information flow through deep convolutional layers, which solves the issues of network degradation and deepens the architecture without the necessity of additional parameters and computation. The generalizability and convergence of the model are improved. When using the same network architecture and classification algorithm in the three sample spaces, the experimental results showed that the performance of the ensemble model was better suited than that of the individual classifier models. Most notably, the E-ResNet-50+NRC model proposed proved better than the other six architectures tested; this model exhibited a higher accuracy, sensitivity, specificity, *F*-value, and MCC, as well as a robust depth and generalizability. Finally, it is noted that the training times were significantly increased; this can be mitigated by the integration of more powerful hardware such as GPUs or cloud computing platforms. Additionally, in the PET sample space, the classification accuracy of all models was relatively high; this is because the PET silhouette contains less information, mainly highlighted information, which accounts for a large contrast—the explanation is not clear.

## 5. Conclusion

In this paper, an E-ResNet-NRC model was proposed and implemented by dividing the sample space based on ensemble learning and using nonnegative representation classification with a ResNet for the classification of medical images of lung tumors. Firstly, the parameters were initialized using transfer learning from a pretrained ResNet. Next, the sample is divided into three different spaces (CT, PET, and PET/CT) according to the different medical imaging techniques. The ResNet extracts the ROI features and uses them to construct feature vectors. Then, an individual classifier, ResNet-NRC, was constructed by employing nonnegative NRC at the fully connected layer. Finally, the ensemble classifier E-ResNet-NRC was achieved by employing relative majority voting. The experimental results showed that the overall classification performance of the proposed E-NRC-ResNet model was better than that of the individual classifier. Its specificity and sensitivity were also higher, while possessing good robustness and generalizability.

## Figures and Tables

**Table 1:** NRC algorithm.

Algorithm: NRC	
1	Input: training sample matrix *X* = [*X*_1_, ⋯., *X*_*k*_] and query sample *y*
2	Normalize each column of matrix *X* and query sample *y* to the unit *L*_2_ norm
3	The encoding vector of *y* on *X* is solved by the NRC model
4	Calculate the coefficient matrix: $\hat{c}=\arg\min_{c}{\left\|y-Xc\right\|}_{2}^{2}s.t.c\geq 0$
5	Calculate residual similarity: $r_{k}={\left\|y-X_{k}{\hat{c}}_{k}\right\|}_{2}$
6	Output label category: *Label*(*y*) = argmin{*r*_*k*_}

**Table 2:** ResNet-50 parameters.

Layers	Output size	Parameter
Conv1	112 × 112	7 × 7, 64, stride 2
Max-Pool	112 × 112	3 × 3, 64, stride 2
Conv2_x	56 × 56	$\left[\begin{array}{c} 1\times 1,64\\ 3\times 3,64\\ 1\times 1,256 \end{array}\right]\times 3$
Conv3_x	28 × 28	$\left[\begin{array}{c} 1\times 1,128\\ 3\times 3,128\\ 1\times 1,512 \end{array}\right]\times 4$
Conv4_x	14 × 14	$\left[\begin{array}{c} 1\times 1,256\\ 3\times 3,256\\ 1\times 1,1024 \end{array}\right]\times 6$
Conv5_x	7 × 7	$\left[\begin{array}{c} 1\times 1,512\\ 3\times 3,512\\ 1\times 1,2048 \end{array}\right]\times 3$
Avg_pool	7 × 7	2048
FC	1 × 1	1000

**Table 3:** Comparison of accuracy, standard deviation, and training times in different models.

	Evaluation index	AlexNet+NNC	AlexNet+Softmax	AlexNet+NRC	ResNet-50+NNC	ResNet-50+Softmax	ResNet-50+NRC
CT	Acc (%)	96.37	98.20	98.80	97.00	98.13	99.07
	SD (%)	1.29	1.14	0.93	1.51	1.34	0.50
	Training time (s)	161.48	164.33	185.91	1176.91	1182.28	1204.95

PET	Acc (%)	99.57	99.50	99.83	99.50	99.63	99.80
	SD (%)	0.75	1.31	0.25	1.31	1.30	0.48
	Training time (s)	146.63	148.77	169.46	1035.85	1036.29	1059.48

PET/CT	Acc (%)	96.60	97.63	97.97	97.53	97.90	98.33
	SD (%)	5.04	3.60	3.37	3.14	2.63	3.01
	Training time (s)	139.25	138.29	162.38	1162.93	1169.76	1187.22

Ensemble	Acc (%)	99.10	98.87	99.43	99.23	99.33	99.57
	SD (%)	2.03	2.28	0.86	1.67	1.45	1.07
	Training time (s)	422.39	420.09	491.20	3080.76	3234.90	3179.38

**Table 4:** Accuracies of different network models and classification algorithms.

Network model	Classification algorithm	CT (%)	PET (%)	PET/CT (%)	Ensemble (%)
AlexNet	NNC	96.37	99.57	96.60	99.10
	Softmax	98.20	99.50	97.63	98.87
	NRC	98.80	99.83	97.97	99.43

ResNet-50	NNC	97.00	99.50	97.53	99.23
	Softmax	98.13	99.63	97.90	99.33
	NRC	99.07	99.80	98.33	99.57

**Table 5:** Comparison of sensitivity results of different network models and classification algorithms.

Network model	Classification algorithm	CT (%)	PET (%)	PET/CT (%)	Ensemble (%)
AlexNet	NNC	99.00	100.00	99.60	99.87
	Softmax	99.20	100.00	98.27	99.27
	NRC	99.07	100.00	98.87	99.53

ResNet-50	NNC	98.80	100.00	99.00	99.93
	Softmax	98.73	100.00	98.80	99.87
	NRC	99.40	100.00	99.33	99.73

**Table 6:** Comparison of specificity results of different network models and classification algorithms.

Network model	Classification algorithm	CT (%)	PET (%)	PET/CT (%)	Ensemble (%)
AlexNet	NNC	93.73	99.13	93.60	98.33
	Softmax	97.20	99.00	97.00	98.47
	NRC	98.53	99.67	97.07	99.33

ResNet-50	NNC	95.20	99.00	96.07	98.53
	Softmax	97.53	99.27	97.00	98.80
	NRC	98.73	99.60	97.33	99.40

**Table 7:** Comparison of *F*-value results of different network models and classification algorithms.

Network model	Classification algorithm	CT (%)	PET (%)	PET/CT (%)	Ensemble (%)
AlexNet	NNC	96.46	99.57	96.70	99.11
	Softmax	98.22	99.50	97.65	98.87
	NRC	98.80	99.83	97.98	99.43

ResNet-50	NNC	97.05	99.50	97.57	99.24
	Softmax	98.14	99.63	97.92	99.34
	NRC	99.07	99.80	98.35	99.57

**Table 8:** Comparison of MCC results of different network models and classification algorithms.

Network model	Classification algorithm	CT (%)	PET (%)	PET/CT (%)	Ensemble (%)
AlexNet	NNC	92.86	99.14	93.37	98.21
	Softmax	96.42	99.00	95.27	97.74
	NRC	97.66	99.67	95.95	98.87

ResNet-50	NNC	94.06	99.00	95.11	98.48
	Softmax	96.27	99.27	95.82	98.67
	NRC	98.14	99.60	96.69	99.13

## Data Availability

The data used to support the findings of this study are available from the corresponding author upon request.
